# Effects of Goreisan in the Perioperative Period of Subthalamic Deep Brain Stimulation in Parkinson's Disease

**DOI:** 10.1002/brb3.70069

**Published:** 2024-10-28

**Authors:** Hiroyuki Kajikawa, Keita Matsuura, Yuichiro Ii, Ken‐ichi Tabei, Naoko Nakamura, Hidehiro Ishikawa, Yamato Nishiguchi, Kana Matsuda, Ken Kagawa, Naoki Ichikawa, Tomohiro Araki, Akihiro Shindo

**Affiliations:** ^1^ Department of Neurology, Graduate School of Medicine Mie University Mie Japan; ^2^ Department of Neurorolgy Suzukakaisei Hospital Mie Japan; ^3^ Department of Neuroimaging and Pathophysiology Mie University School of Medicine Mie Japan; ^4^ School of Industrial Technology, Advanced Institute of Industrial Technology Tokyo Metropolitan Public University Corporation Tokyo Japan; ^5^ Department of Dementia Prevention and Therapeutics Mie University Graduate School of Medicine Mie Japan; ^6^ Department of Neurosurgery Suzukakaisei Hospital Mie Japan

**Keywords:** brain edema, deep brain stimulation, Goreisan, Parkinson's disease

## Abstract

**Introduction:**

Patients with Parkinson's disease (PD) may benefit from deep brain stimulation (DBS). Perifocal brain edema sometimes occurs after DBS surgery, but it is transient and does not affect the final prognosis. Transient deterioration of cognitive function has been reported in patients with frontal edema in the first postoperative week. This study aimed to investigate the effect of Goreisan in preventing edematous changes after DBS and determine the influence of edema on cognition.

**Methods:**

We included 29 patients with PD who underwent bilateral subthalamic nucleus (STN) DBS and who were divided into 2 groups: those using (11 patients) and those not using Goreisan (18 patients). At 1 week postoperatively, all patients underwent magnetic resonance imaging. We measured the volume of edema either in the frontal white matter or STN on fluid‐attenuated inversion recovery (FLAIR) images. Finally, brain edema, motor function, and cognitive function were compared between the groups with and without Goreisan.

**Results:**

In the FLAIR image 1 week postoperatively, the average postoperative frontal subcortical edema (FE) volume of the group with Goreisan was significantly lower than that without Goreisan (2249 ± 2186 mm^3^, 6261 ± 7213 mm^3^, respectively, *p* = 0.023). Multivariate analysis with age, preoperative Mini‐Mental State Examination (MMSE) score, FE, and peri‐STN edema (SE) as factors, and MMSE at 1 week postoperatively as the dependent variable showed that preoperative MMSE score and SE were significant as associated factors.

**Conclusions:**

FE after DBS surgery may be alleviated using Goreisan. SE and preoperative MMSE scores were associated with MMSE scores 1 week postoperatively.

**Trial Registration:**

Not applicable

## Graphical abstract

One week after deep brain stimulation surgery, edema occurred frequently around the leads. The frontal edema volume had significantly less edema in the group that used Goreisan. MMSE and MoCA‐J scores at 1 week were significantly worse in the presence of edema in both frontal and peri‐STN than preoperatively.

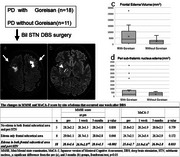

## 1 Introduction

Deep brain stimulation (DBS) is an established therapy for advanced Parkinson's disease (PD) (Krack et al. 2003). However, patients who undergo DBS may experience side effects in the perioperative period, such as headaches, seizures, recall difficulty, and postoperative deterioration of cognitive function (Borellini et al. 2019). Cerebral edema is common after DBS surgery. Borellini et al. (2019) reported brain edema in all 19 consecutive patients who underwent magnetic resonance imaging (MRI) at 7–20 days postoperatively. Our previous study reported brain edema in 77% of the 13 consecutive patients who underwent MRI at 1 week postoperatively (Nishiguchi et al. 2022). Conversely, the incidence of cerebral edema was 14.7%, which was assessed using MRI at 6 weeks postoperatively (Whiting et al. 2018). Furthermore, no brain edema was observed on MRI at 12 weeks postoperatively (Nishiguchi et al. 2022). The detection rate on MRI may change depending on the interval from the surgery. Our previous study suggested a relationship between frontal edema and worsening cognitive function at the 1‐week postoperative DBS evaluation (Nishiguchi et al. 2022). This worsening of cognitive function, although transient, cannot be overlooked for safer treatment (Borellini et al. 2019; Nishiguchi et al. 2022; Whiting et al. 2018).

It is hypothesized that post‐DBS brain edema may be attributed to vasogenic edema resulting from mechanical microtrauma and microhemorrhage associated with DBS. Additionally, age‐related immune dysregulation has been proposed as a potential contributing factor (Giordano et al. 2023). Moreover, there are reports indicating that Aquaporin 4 (AQP4) is upregulated in models of traumatic brain injury (Ren et al. 2013). AQP4, an astrocyte‐expressed water channel, plays a crucial role in regulating water transfer across the blood–brain barrier, cerebrospinal fluid volume, and hormone secretion (Nagelhus and Ottersen 2013; Salman et al. 2021). During cerebral edema formation, AQP4‐mediated water transfer from blood vessels to the brain accelerates (Filippidis, Carozza, and Rekate 2016; Kozono et al. 2003; Luo et al. 2020; Papadopoulos et al. 2004; Papadopoulos and Verkman 2007; Xiong et al. 2021). Notably, cerebral edema caused by acute water intoxication formation in AQP4 knockout mice is milder (Manley et al. 2000). Therefore, inhibiting AQP4 function could help suppress cerebral edema formation. Goreisan, a traditional diuretic, is an approved Kampo medicine in Japan used to treat headaches, dizziness, and diarrhea (Nakano et al. 2018). Clinically, Goreisan is effective in treating headaches and chronic subdural hematoma (Fujisawa et al. 2021; Katsuki et al. 2021; Ohsawa et al. 2021; Yasunaga 2015). Goreisan inhibits upregulation of AQP4 in rodent models of acute cerebral ischemia (Nakano et al. 2018; Yano et al. 2017). Hence, the mechanism of how Goreisan treats headaches could involve the regulation of water dynamics. However, the precise effect of Goreisan on water permeability through AQP4 remains unknown. Thus, we hypothesized that Goreisan could control postoperative edema after DBS surgery.

This study aimed to investigate whether Goreisan can suppress edema after DBS surgery and determine the effects of brain edema on cognition.

## 2 Materials and Methods

### 2.1 Participants

We retrospectively evaluated 29 patients with PD who underwent bilateral subthalamic (bilateral subthalamic nucleus [STN]) DBS surgery between January 2018 and September 2020. Moreover, postoperative MRI was performed at 1 week and 3 months postoperatively. Of the 29 patients, 11 used Goreisan beginning postoperatively.

### 2.2 Surgery Procedures

All patients underwent bilateral electrode placement for STN DBS. Electrodes (Vercise Cartesia Directional Lead, Boston Scientific, Natick, MA, USA) were implanted under local anesthesia by using a Leksell stereotactic frame (Elekta Instruments AB, Stockholm, Sweden) and anatomical (MRI and computed tomography [CT]) and physiological targeting. Electrodes were considered correctly located in the target region based on microelectrode recordings. Impulse generators (Vercise Gevia or Genus R16, Boston Scientific, Natick, MA, USA) were implanted and connected during a second surgical procedure on the same day (Matsuura et al. 2019).

### 2.3 MRI Data Acquisition and Analysis

All postoperative MRI images were obtained using a 1.5‐T MR unit (Ingenia; Philips Healthcare, Best, The Netherlands). The pulse sequence used for fluid‐attenuated inversion recovery (FLAIR) image was a three‐dimensional turbo spin‐echo (TSE) technique with repetition, echo, and inversion times of 4800, 421, and 1660 ms, respectively, as well as TSE factor of 178, field of view of 260 mm, matrix size of 236 × 236 (pixel size of 1.10 × 1.10 mm^2^), slice thickness of 1.14 mm, recon voxel size of 0.51 × 0.51 × 0.57, 1‐averaged, 305 slices, and acquisition time of 6 min 24 s. These conditions conform to the officially accepted values in Japan for MRI examinations after DBS surgery.

We used FLAIR to calculate the volume of brain edema. For frontal subcortical edema (FE) and peri‐STN edema (SE), we initially reconstructed the axial images from sagittal FLAIR with 5‐ and 2‐mm thickness, respectively. Second, we determined the high signal area of each slice of FLAIR images by freehand and measured its area. Third, we calculated volume using the area and thickness (Nishiguchi et al. 2022). All edema volume measurements were performed in a blinded manner by an experienced rater (YN), who had demonstrated excellent agreement of inter‐/intra‐rater intraclass correlation coefficients for FE (0.977/0.998) and SE (0.929/0.971) in our previous study (Nishiguchi et al. 2022).

### 2.4 Patient Data Analysis

We performed assessments including the Japanese version of the Movement Disorder Society Revision of the Unified PD Rating Scale (MDS‐UPDRS), Mini‐Mental State Examination (MMSE), Japanese version of Montreal Cognitive Assessment (MoCA‐J), Raven's colored progressive matrices (RCPM), pareidolia test, apathy scale, and the Center for Epidemiologic Studies Depression Scale (CES‐D) for all patients before DBS (i.e., baseline) and at 10 days and 3 months postoperatively (Kashihara et al. 2014; Folstein, Folstein, and McHugh 1975; Fujiwara et al. 2010; Uchiyama et al. 2012; Raven 1941; Lewinsohn et al. 1997; Partington and Leiter 1949). We calculated the l‐dopa equivalent daily dose (LEDD) as follows: 100‐mg l‐dopa/decarboxylase inhibitor = 1‐mg pramipexole = 5‐mg ropinirole = 3.3 mg/day rotigotine = 1.5‐mg cabergoline = 1‐mg pergolide = 1‐mg amantadine hydrochloride = 75‐mg l‐dopa/decarboxylase inhibitor with entacapone = 10‐mg selegiline = 1‐mg rasagiline = 83‐mg l‐dopa/decarboxylase inhibitor with istradefylline = zonisamide use = one time use of trihexyphenidyl (Jost et al. 2023).

### 2.5 Statistical Analysis

For statistical analyses, Mann–Whitney *U*‐tests were used to compare the Hoehn–Yahr stage, MDS‐UPDRS, MMSE, MoCA‐J, RCPM, pareidolia test, apathy scale, and CES‐D. Fisher's exact tests were used for categorical data. We used paired *t*‐tests to compare LEDD, l‐dopa dosage, and FE and SE volumes between patients with and without Goreisan. We used Friedman and Bonferroni tests to determine differences between MMSE and MoCA‐J scores preoperatively and 1 week and 12 weeks postoperatively. We performed multivariate regression analysis using a generalized linear model. All statistical analyses were performed using Statistical Package for Social Sciences (SPSS) version 29 (IBM, Armonk, NY, USA). An α level of 0.05 was used to determine the significance of all statistical tests.

## 3 Results

In the group not using Goreisan, the mean age and disease duration were 63.3 ± 9.8 and 13.7 ± 7.0 years, respectively, including 7 men and 11 women. In the group using Goreisan, the mean age and disease duration were 64.9 ± 5.6 and 13.6 ± 14.7 years, respectively, including 4 men and 7 women. Smoking, hypertension, hyperlipidemia, history of cerebrovascular disease, PD severity, drug use, cognitive function, depression scale, and apathy scale were not significantly different between the two groups, except for the frequency of DA use and HY at off‐state (Table 1).

**TABLE 1:** Demographic data and overall result.

	All patients	Without Goreisan	With Goreisan	*p* value
Age	63.8 ± 8.4	63.3 ± 9.8	64.9 ± 5.6	0.735
Numbers (M/F)	29 (11/18)	18 (7/11)	11 (4/7)	0.892
Disease duration (years)	13.7 ± 10.4	13.7 ± 7.0	13.6 ± 14.7	0.983
DBS target	All STN	All STN	All STN	
Smoking (%)	28	22	36	0.408
Hypertension (%)	31	39	18	0.242
Hyperlipidemia (%)	10	11	9.1	0.862
PH of vascular disease (%)	10	5.6	18	0.279
Hoehn–Yahr stage (on state)	2.7 ± 0.8	2.8 ± 0.9	2.5 ± 0.7	0.580
Hoehn–Yahr stage (off state)	4.1 ± 0.7	4.4 ± 0.5	3.7 ± 0.8	**0.039**
MDS‐UPDRS Part I	9.4 ± 4.5	9.4 ± 4.7	9.3 ± 4.2	0.877
MDS‐UPDRS Part II	11.4 ± 6.5	10.9 ± 6.4	12.1 ± 7.0	0.842
MDS‐UPDRS Part III on	19.8 ± 11.4	17.8 ± 12.5	23.0 ± 9.0	0.134
MDS‐UPDRS Part III off	39.9 ± 17.0	40.2 ± 17.1	39.4 ± 17.8	0.912
MDS‐UPDRS Part IV	8.2 ± 4.8	9.6 ± 4.8	6.0 ± 4.0	0.068
LEDD (mg)	952 ± 327	1013 ± 237	853 ± 432	0.258
l‐Dopa (mg)	481 ± 164	489 ± 161	468 ± 176	0.506
DA (use rate; %)	83	94	64	**0.033**
Entacapone (use rate; %)	69	72	64	0.628
MAO‐B inhibiter (use rate; %)	35	33	36	0.868
Zonisamide (use rate; %)	35	33	36	0.868
Istradefylline (use rate; %)	31	39	18	0.242
MMSE	28.6 ± 1.6	28.2 ± 1.8	29.2 ± 1.0	0.220
MoCA‐J	25.4 ± 2.7	25.3 ± 2.5	25.6 ± 3.0	0.740
RCPM	30.3 ± 3.8	30.3 ± 4.0	30.3 ± 3.5	0.912
Time of RCPM	368 ± 248	316 ± 129	452 ± 361	0.155
Pareidolia test (%)	3.1 ± 8.6	3.7 ± 10.7	2.0 ± 3.5	0.774
FAB	16.0 ± 1.8	15.9 ± 2.1	16.1 ± 1.3	0.877
Apathy scale	13.7 ± 5.9	12.9 ± 6.0	15.0 ± 5.8	0.521
CES‐D	17.0 ± 8.9	15.6 ± 8.4	19.4 ± 9.6	0.317

*Note*: The bold letter indicates *p* < 0.05.

Abbreviations: DBS, deep brain stimulation; LEDD, levodopa equivalent daily dose; MDS‐UPDRS, Movement Disorder Society‐Sponsored Revision of the Unified Parkinson's Disease Rating Scale; MMSE, Mini‐Mental State Examination; MoCA‐J, Japanese version of Montreal Cognitive Assessment; RCPM, Raven's Colored Progressive Matrices; STN, subthalamic nucleus.

**FIGURE 1:**
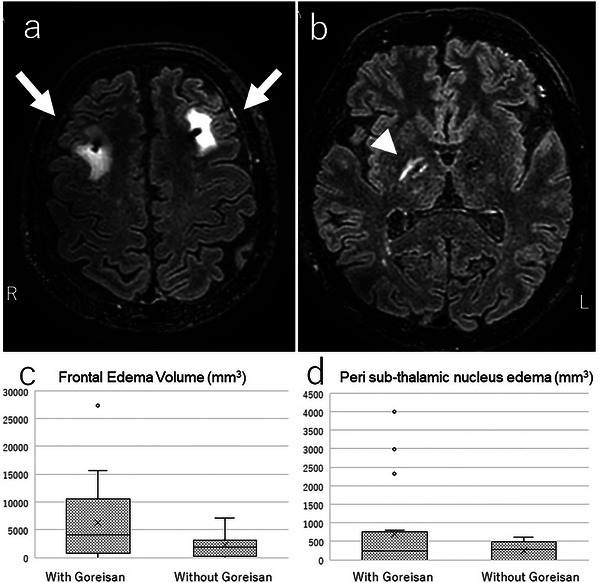
Representative cases of post‐DBS edema (65‐year‐old patient): (a) frontal edema (arrow); (b) perisubthalamic nucleus edema (arrowhead); (c and d) These are expressed as box‐and‐whisker plots—(c) frontal edema and (d) perisubthalamic nucleus edema volume. DBS, deep brain stimulation.

At 1 week postoperatively, cerebral edema was observed in 24 patients (83%) on MRI, including 18 cases with both FE and SE and 6 with FE alone (Table 2). On the basis of radiological features, the increased FLAIR signal of the white matter around the track was consistent with vasogenic edema. The mean FE volume in the group with Goreisan was significantly lower than that without Goreisan (2249 ± 2186 vs. 6261 ± 7213 mm^3^, *p* = 0.039). The mean SE volume with and without Goreisan was not significantly different (247 ± 251 vs. 701 ± 1169 mm^3^, *p* = 0.129) (Table 2 and Figure 1). The comparison of MRI at 1 week postoperatively showed no significant difference in the number of patients with FE ≥ 3000 mm^3^ between the two groups (11/18 [61%] vs. 3/11 [27%], *p* = 0.058). The MDS‐UPDRS, MMSE, MoCA‐J, RCPM, pareidolia test, apathy scale, or CES‐D scores were not significantly different between both groups at 1 week postoperatively, except for the score change from the baseline of MDS‐UPDRS part IV (Table 2).

**TABLE 2:** Result at 1 week after deep brain stimulation (DBS).

	Without Goreisan	With Goreisan	*p* value
Number	18	11	—
Edema in both the frontal subcortical area and peri‐STN	12	6	0.514
Edema only frontal subcortical area	3	3	0.664
Edema only peri‐STN	0	0	—
No edema in both the frontal subcortical area and peri‐STN	3	2	0.917
Frontal subcortical edema (mm^3^)	6261 ± 7213	2249 ± 2186	**0.039**
The number of frontal subcortical edema ≥ 3000 mm^3^	11 (61%)	3 (27%)	0.058
Peri‐STN edema (mm^3^)	701 ± 1169	247 ± 251	0.129
The number of peri‐STN edema ≥ 1000 mm^3^	3	0	0.102
MDS‐UPDRS			
Part I	5.2 ± 3.2	6.3 ± 3.8	0.387
Part II	9.3 ± 7.4	10.8 ± 6.7	0.521
Part III	17.8 ± 13.8	18.6 ± 11.6	0.674
Part IV	3.3 ± 3.3	3.3 ± 3.0	1.000
Part I: change from baseline	− 4.3 ± 5.0	− 3.0 ± 4.8	0.499
Part II: change from baseline	− 1.7 ± 6.1	− 1.3 ± 3.6	0.829
Part III: change from baseline	− 0.1 ± 14.9	− 4.4 ± 10.6	0.373
Part IV: change from baseline	− 6.3 ± 4.4	− 2.7 ± 3.8	**0.031**
LEDD (mg)	528 ± 153	614 ± 310	0.328
l‐Dopa (mg)	313 ± 79	350 ± 184	0.452
MMSE	27.3 ± 2.6	27.8 ± 2.1	0.680
MMSE score change from baseline	− 1.4 ± 2.2	− 0.9 ± 2.1	0.615
MoCA‐J	24.3 ± 3.7	24.5 ± 2.9	0.803
MoCA‐J score change from baseline	− 1.2 ± 2.6	− 0.9 ± 4.1	0.512
RCPM	28.6 ± 5.0	28.8 ± 5.0	0.892
Time of RCPM (s)	313 ± 159	371 ± 147	0.339
Time of RCPM change from baseline (s)	2.9 ± 81.9	81.1 ± 243.5	0.218
FAB	15.7 ± 1.8	15.5 ± 2.2	0.947
FAB score change from baseline	− 0.2 ± 2.0	− 0.5 ± 2.7	0.982
Pareidolia test (%)	0.8 ± 2.1	2.0 ± 6.8	0.808
Apathy scale	12.7 ± 7.8	15.5 ± 5.2	0.188
CES‐D	8.7 ± 10.4	9.2 ± 5.4	0.276

*Note*: The bold letter indicates *p* < 0.05.

Abbreviations: CES‐D, The Center for Epidemiologic Studies Depression Scale; DA, dopamin agonist; LEDD, levodopa equivalent daily dose; MDS‐UPDRS, Movement Disorder Society‐Sponsored Revision of the Unified Parkinson's Disease Rating Scale; MMSE, Mini‐Mental State Examination; MoCA‐J, Japanese version of Montreal Cognitive Assessment; RCPM, Raven's Colored Progressive Materices; STN, subthalamic nucleus.

All patients had no cerebral edema on MRI at 12 weeks postoperatively. The MDS‐UPDRS, MMSE, MoCA‐J, RCPM, pareidolia test, apathy scale, or CES‐D scores were not significantly different between both groups at 12 weeks postoperatively, except for the MDS‐UPDRS part III score (Table 3).

**TABLE 3:** Result at 12 weeks after deep brain stimulation (DBS).

	Without Goreisan	With Goreisan	*p* value
Number	18	11	—
The case number of edema	0	0	—
MDS‐UPDRS			
Part I	5.2 ± 3.2	5.1 ± 2.1	0.707
Part II	5.2 ± 3.2	7.1 ± 4.7	0.363
Part III on	7.3 ± 5.6	13.1 ± 8.2	**0.035**
Part III off	12.1 ± 11.0	15.9 ± 10.2	0.317
Part IV	3.1 ± 3.0	2.3 ± 2.4	0.642
Part I: change from baseline	− 4.2 ± 4.4	− 4.2 ± 4.9	0.982
Part II: change from baseline	− 5.8 ± 6.6	− 5.0 ± 5.6	0.738
Part III on: change from baseline	− 10.6 ± 13.9	− 9.9 ± 11.7	0.894
Part III off: change from baseline	− 28.1 ± 19.0	− 23.5 ± 15.5	0.480
Part IV: change from baseline	− 6.4 ± 4.7	− 3.7 ± 3.8	0.103
LEDD (mg)	556 ± 194	581 ± 342	0.802
l‐Dopa (mg)	314 ± 89	318 ± 209	0.939
MMSE	28.3 ± 1.7	28.5 ± 2.0	0.550
MMSE score change from baseline	0.1 ± 1.6	− 0.6 ± 2.0	0.204
MoCA‐J	26.2 ± 2.7	25.8 ± 2.8	0.707
MoCA‐J score change from baseline	0.9 ± 1.7	0.2 ± 2.6	0.276
RCPM	28.9 ± 4.5	29.9 ± 4.5	0.465
Time of RCPM (s)	250 ± 94	317 ± 96	0.079
Time of RCPM change from BL (s)	67 ± 70	135 ± 303	0.359
FAB	16.8 ± 1.2	16.1 ± 1.6	0.238
FAB score change from baseline	0.9 ± 2.3	0.0 ± 1.7	0.642
Pareidolia test (%)	0.8 ± 1.7	0	0.340
Apathy scale	12.6 ± 6.3	14.8 ± 7.5	0.256
CES‐D	9.2 ± 8.5	10.5 ± 11.9	0.947

*Note*: The bold letter indicates *p* < 0.05.

Abbreviations: CES‐D, The Center for Epidemiologic Studies Depression Scale; DA, dopamin agonist; LEDD, levodopa equivalent daily dose; MDS‐UPDRS, Movement Disorder Society‐Sponsored Revision of the Unified Parkinson's Disease Rating Scale; MMSE, Mini‐Mental State Examination; MoCA‐J, Japanese version of Montreal Cognitive Assessment; RCPM, Raven's Colored Progressive Materices; STN, subthalamic nucleus.

**FIGURE 2:**
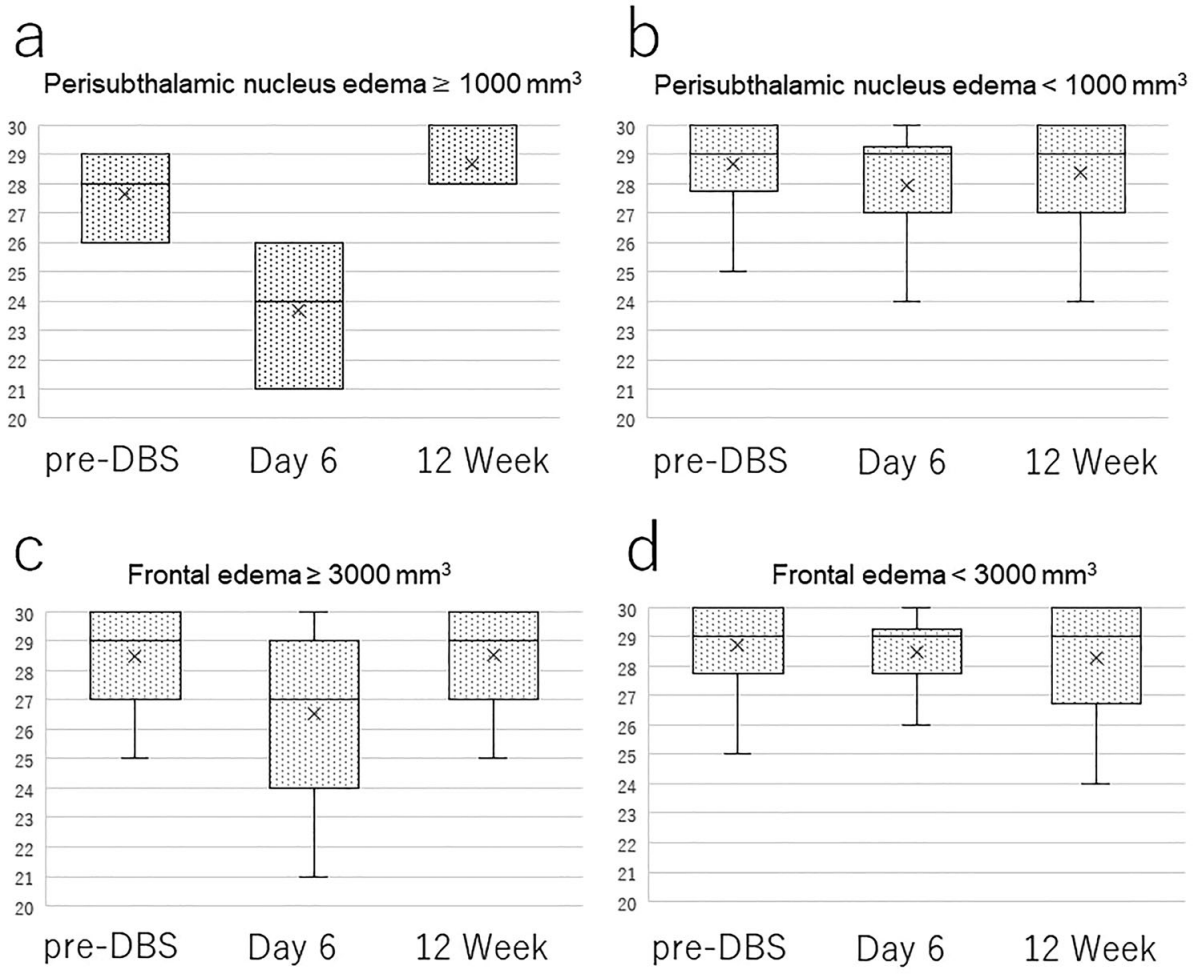
MMSE scores are expressed as box‐and‐whisker plots: (a) perisubthalamic nucleus edema volume ≥ 1000 mm^3^, (b) perisubthalamic nucleus edema volume < 1000 mm^3^, (c) frontal edema volume ≥ 3000 mm^3^, and (d) frontal edema volume < 3000 mm^3^. DBS, deep brain stimulation.

In this series, three patients with SE ≧ 1000 mm^3^ who did not use Goreisan had lower MMSE scores at 1 week postoperatively than preoperatively and at 12 weeks postoperatively (Figure 2) (no statistical processing due to the small number of cases). At 1 week postoperatively, the MMSE score between 14 patients (including 11 who used Goreisan) with FE > 3000 mm^3^ and 15 cases with FE < 3000 mm^3^ had no significant difference (26.7 ± 2.9 vs. 28.2 ± 1.7, *p* = 0.110) (Figure 2). The MMSE and MoCA‐J scores preoperatively and at 1 week and 12 weeks postoperatively were not significantly different in the group without FE and SE, as well as the group with only FE, but were significantly different in the group with SE (Table 4). We performed a multivariate analysis of three factors for the MMSE score at 1 week postoperatively: age, preoperative MMSE score, and FE and SE volumes. The standardized partial regression coefficients for age, preoperative MMSE score, FE, and SE were 0.978 (95% confidence interval [CI], 0.906–1.056; *p* = 0.574), 1.965 (95% CI, 1.281–3.015; *p* = 0.002), 1.000 (95% CI, 1.000–1.000; *p* = 0.09), and 0.998 (95% CI, 0.997–0.999; *p* = 0.001), respectively (Table 5).

**TABLE 4:** The changes in Mini‐Mental State Examination (MMSE) and Japanese version of Montreal Cognitive Assessment (MoCA‐J) scores by site of edema that occurred 1 week after deep brain stimulation (DBS).

		MMSE score at pre				MoCA‐J			
	*n*	pre	1 week	3 months	*p* value	pre	1 week	3 months	*p* value
No edema in both the frontal subcortical area and peri‐STN	5	28.2 ± 2.2	28.2 ± 1.3	28.2 ± 1.8	0.838	25.8 ± 3.2	26.2 ± 1.9	26.0 ± 2.5	0.779
Edema only frontal subcortical area	6	29.0 ± 1.1	28.7 ± 1.5	28.0 ± 2.8	0.646	24.7 ± 2.5	24.2 ± 3.4	26.2 ± 2.9	0.172
Edema in both the frontal subcortical area and peri–STN	18	28.6 ± 1.6	26.9 ± 2.8^ab^	28.6 ± 1.5	< 0.001	25.6 ± 2.7	23.9 ± 3.6^b^	26.0 ± 2.8	0.033

*Note*: a significant difference from the pre (a) and 3 month (b) groups, the Bonferroni test (*p* < 0.05).

Abbreviation: STN, subthamic nucleus.

**TABLE 5:** Factors on post–deep brain stimulation (DBS) Mini‐Mental State Examination (MMSE) score were evaluated with multivariate regression analysis.

	SPRC	95% CI	*p* value
Age	0.574	0.906–1.056	0.184
Preoperative MMSE score	1.965	1.281–3.015	< 0.001
Frontal edema volume	1.000	1.000–1.000	0.09
Perisubthalamic nucleus edema	0.998	0.997–0.999	0.001

Abbreviation: SPRC, standardized partial regression coefficient.

## 4 Discussion

The brain edema rate after DBS was 5.4%–100%. Previous reports have used CT or MRI, and the timing of the imaging varied. In this study, all MRI images were obtained at 6 days and 3 months postoperatively. MRI at 1 week after DBS showed brain edema in 24 of 29 cases (83%), but edema resolved at 3 months, which was consistent with our previous study (Nishiguchi et al. 2022).

This study showed that the mean FE volume in patients using Goreisan was significantly smaller than those not using Goreisan, indicating that Goreisan may reduce postoperative brain edema in DBS, which may be due to the inhibition effect of AQP4 in Goreisan (Nakano et al. 2018; Yano et al. 2017).

The Goreisan group showed a more remarkable improvement in MDS‐UPDRS part IV score at 1 week postoperatively, which may be because the SE volume in patients using Goreisan tended to be lesser than those not using Goreisan, consistent with our previous finding that more severe SEs lead to a more substantial lesioning effect (Nishiguchi et al. 2022). However, the SE difference with and without Goreisan was not significant, so further case accumulation and investigation are necessary. Another factor to consider is whether the DBS leads pass near the globus pallidus internus (GPi); edema over the GPi may directly suppress dyskinesia and thus improve UPDRS IV (Fan et al. 2020). This issue must also be addressed in the future, as we were not able to examine the detailed site of edema in this study.

At 12 weeks postoperatively, brain edema resolved on MRI. The MDS‐UPDRS part III score at 12 weeks postoperatively was significantly different between both groups, but the change from baseline showed no difference. Therefore, similar to previous reports, we showed that perioperative edema did not affect the final outcome (Borellini et al. 2019; Nishiguchi et al. 2022; Whiting et al. 2018). However, long‐term prognosis has not been investigated, and we consider this an issue for future research.

On the basis of our previous report, cognition declined temporarily in the FE+ group (Nishiguchi et al. 2022). Moreover, multivariate analysis showed that SE was significantly associated with MMSE decline at 1 week postoperatively. In our previous report (Nishiguchi et al. 2022), we included all SE+ in FE+, leading to the conclusion that FE+ may have a significant impact on cognitive decline. In the present study, however, the group with only FE+ did not show a significant decrease in MMSE scores after 1 week (Table 4). The STN is close to the thalamus, and DBS leads pass nearby. The SE extends to the thalamus, so edema of the thalamus may be associated with cognitive decline (Hwang et al. 2017).

In a previous report, Saitoh et al. (2019) reported brain edema in 6 of 15 patients after 3–10 days postoperative MRI; in the present study, in which MRI was performed 7 days after DBS, brain edema was seen in 24 of 29 patients. In the study by Borellini et al. (2019), in which MRI was performed 7–20 days postoperatively, all 19 patients had brain edema. Furthermore, in the study by Whiting et al. (2018), in which an MRI was performed 2 months after DBS, edema was seen in 15 of 102 patients. These results suggest that brain edema after DBS may be most pronounced between 1 (Whiting et al. 2018) and 3 weeks, but no longitudinal studies have been conducted, and this is an issue for the future. In addition, our study is the only one to evaluate cognitive function in detail. Prospective, long‐term follow‐up is needed to clarify the clinical impact, including motor and cognitive function of brain edema after DBS.

Previous and present results did not show any long‐term effect postoperatively; however, whether the effect lasts long‐term is uncertain, and we believe that this should be investigated further in future studies (Whiting et al. 2018; Nishiguchi et al. 2022; Borellini et al. 2019).

The present study has several limitations. First, this was a retrospective study, and Goreisan allocation was not randomized. Thus, future studies should be prospective and based on the random assignment of more cases. Second, the duration of the actual brain edema persisting on MRI is uncertain, and more frequent MRI follow‐up would be desirable.

## 5 Conclusion

Goreisan may alleviate FE after DBS surgery. The SE and preoperative MMSE scores were associated with MMSE at 1 week postoperatively.

## Author Contributions

**Hiroyuki Kajikawa**: writing–original draft, investigation, conceptualization, resources. **Keita Matsuura**: conceptualization, investigation, funding acquisition, methodology, validation, visualization, writing–review and editing, project administration, resources. **Yuichiro Ii**: conceptualization, investigation, validation, writing–review and editing, supervision. **Ken‐ichi Tabei**: software, formal analysis. **Naoko Nakamura**: investigation, conceptualization. **Hidehiro Ishikawa**: investigation. **Yamato Nishiguchi**: conceptualization, investigation, methodology, validation, visualization, formal analysis, data curation. **Kana Matsuda**: investigation. **Ken Kagawa**: conceptualization, investigation. **Naoki Ichikawa**: investigation, methodology. **Tomohiro Araki**: investigation, methodology. **Akihiro Shindo**: writing–review and editing, project administration, supervision, funding acquisition.

## Ethics Statement

The Ethical Review Board of Mie University Hospital (H2019‐134) and Suzuka Kaisei Hospital (2019‐11) approved our study.

## Consent

Informed consent was provided by all patients after enrollment in the study.

## Conflicts of Interest

The authors declare no conflicts of interest.

### Peer Review

The peer review history for this article is available at https://publons.com/publon/10.1002/brb3.70069.

## Permission to Reproduce Material from Other Sources

Not applicable.

## Data Availability

The data generated and analyzed in this study are available from the corresponding author upon reasonable request.
